# Composition and Biomass of Aquatic Vegetation in the Poyang Lake, China

**DOI:** 10.1155/2017/8742480

**Published:** 2017-02-09

**Authors:** Wei Du, Ziqi Li, Zengxin Zhang, Qiu Jin, Xi Chen, Shanshan Jiang

**Affiliations:** ^1^Joint Innovation Center for Modern Forestry Studies, College of Biology and the Environment, Nanjing Forestry University, Nanjing 210037, China; ^2^Middle School Affiliated to Hubei University, Wuhan 430062, China; ^3^State Key Laboratory of Hydrology-Water Resources and Hydraulics Engineering, Hohai University, Nanjing 210098, China

## Abstract

The distribution of aquatic vegetation and associated community diversity and biomass in the Poyang Lake were investigated. The results showed that (1) 43 species of aquatic vascular plants were found in the Poyang Lake watershed which belonged to 22 families; (2) the vegetation of the Poyang Lake scattered in different areas which could be divided into 31 major plant communities and 5 plant zones including amphibian, emergent, floating-leaved, submerged, and floating input; (3) there were 67 aquatic plants in the lake area, and the standing stock (fresh weight) was 1519.41 t. The number of amphibians was the dominant plant species in the Poyang Lake, and the quantity and percentage of amphibians were predominant, which was far more than the other three life forms.

## 1. Introduction

Aquatic plants are an important component of lake ecosystems and are often regarded as indicators of lake environmental changes; they play an important role in maintaining the structure and function of lake ecosystems [1, 2]. Aquatic plants are the primary producers of aquatic ecosystems, being a kind of food source of many kinds of fish and other aquatic animals [3, 4]. They can regulate the lake water body, degrade various pollutants, and improve the transparency [5, 6]. In addition, aquatic plants can provide habitat to many organisms and increase the spatial niche of aquatic ecosystems [7, 8]. Therefore, it is important to know the distribution of aquatic plants and their communities to analyze the status of aquatic plants in the lake area.

Vegetation is an important component of wetland ecosystems, and biomass can quantify the contribution of wetland vegetation to carbon sinks and carbon sources [9]. Biomass estimation of wetlands plays an important role in understanding dynamic changes of the wetland ecosystem [10]. In previous studies, biomass estimation using indirect and direct methods has been conducted. For example, remote sensing technology and radar data have been widely used to estimate the biomass in recent years [2, 11]. Han et al. [11] investigated four decades of winter wetland changes in the Poyang Lake based on Landsat observations between 1973 and 2013. Luo et al. [2] revealed the distribution of aquatic vegetation types in Taihu Lake, China, by using a series of remotely sensed images with a resolution of 30 m (HJ-CCD and Landsat TM). Shen et al. [9] retrieved vegetation biomass in the Poyang Lake wetland by using polarimetric RADARSAT-2 data. These indirect methods offer the ability to continuously monitor growth and phenology on the same individuals over large areas but depend on the existence of strong relationships between the predictor variables and plant biomass [12–14]. Therefore, ground investigation of biomass is also very essential.

Poyang Lake, the largest freshwater lake in China, is well known for its ecological importance as a wetland system [9, 11]. The Poyang Lake region provides significant environmental benefits, such as supplying water resources and maintaining carbon storage and biodiversity [15, 16]. Guan et al. [17] pointed out that aquatic vegetation of Poyang Lake is very rich in number of species. The vegetation distributes in about 2262 km^2^ and accounts for 80.8% area of the lake. Jian et al. [18] studied the distribution area and biomass of Poyang Lake beach vegetation in the years of 1999 and 2000, and they found there were 28 families, 56 genera, 95 species, and 3 varieties. In April, 2000, they found that the total biomass of beach vegetation was 3.81 × 10^6^ tons (fresh weight), with an average biomass of 3736 g·m^−2^ (fresh weight). Peng et al. [19] studied the association type diversity, community species diversity, community coverage, and biomass of aquatic plants in the fresh water lakes of the Poyang Plain District of China in 2001, and they found 42 associations of aquatic plants in the lakes, which include 11 amphibian, 6 emergent, 11 leaf-floating, and 14 submerged associations. Among all the associations,* Carex cinerascens* Ass. and* Zizania latifolia* Ass. possess the highest coverage and highest biomass, respectively. Although some survey of aquatic plants in the Poyang Lake has been conducted, ground investigation of aquatic plants in Poyang Lake was very rare in recent years.

In this paper, the species and biomass of aquatic plants in the Poyang Lake were investigated based on ground survey data in September, 2013. And the objective of this study is to investigate the composition of aquatic plants and community types and habitats of major aquatic plants in the Poyang Lake. This study will provide information for the monitoring and conservation of aquatic plant in the Poyang Lake.

## 2. Data and Methodology

### 2.1. Study Area and Data

Poyang Lake is located on the southern bank of the lower Yangtze reach (28° 22′~29° 45′N and 11° 47′~116° 45′E). It has five main tributary rivers: Ganjiang River, Fuhe River, Xinjiang River, Raohe River, and Xiushui River, and several smaller rivers. The basin area of the five rivers is 162,200 km^2^, occupying 9% of Yangtze River basin. The climate is characterized as a subtropical, humid, monsoon climate with a 1620 mm mean annual precipitation and an annual average temperature of approximately 17°C [20].

The environmental conditions of the Poyang Lake are very suitable for the growth and reproduction of aquatic plants. Aquatic plants can provide habitat for many organisms, improve habitat diversity, and increase the spatial niche of aquatic ecosystem [21]. But the annual water level of the Poyang Lake changes greatly, especially in the flood season (April to November) and dry season (December to March). The characteristics of periodical variation of water level in the Poyang Lake might lead to the conversion of the lake beach and grassland.

### 2.2. Methodology

The species, community structure, coverage, and biomass of the Poyang Lake were investigated from September 7, 2013, to September 14, 2013. GPS and lake electronic map were used to set sampling points, and the samples of aquatic plants were collected at each sampling site. The category of aquatic plants in the Poyang Lake was defined according to the reference of Cook [22]. Division of “cluster” is based on the principle of dominant species, which is named as the name of the group. If a cluster is with two or more dominant species, different dominant species of the same layer were connected with “+,” and the dominant species of different layers were connected with “−.” At the sampling sites, amphibians, emergent plants, floating-leaved plants, and floating input plants were directly observed and recorded. Submerged plants were preliminarily identified with water sickle after collecting water and the characters of flowers, leaves, and fruits of each plant as well as field growth photos and accurate identification of aquatic plant species and genus [23]. For amphibian and emergent plant clusters, 6 samples of 2 m × 2 m were set randomly within 500 m^2^ of each sampling point, and the total biomass (fresh quality) of the aerial parts of all the plants was measured and the biomass per unit area was calculated. For plants with large plant biomass, such as* Phragmites communis *Trin., we could estimate the number of plants in the quadrats. After selecting the representative fresh weight of the plants, we could calculate the standing amount of the plants. As the species with smaller biomass, such as* Alternanthera philoxeroides *(Mart.) Griseb., all the plants in the quadrats were collected and their fresh weight was calculated, and the mean values were calculated after several measurements of plant height. The floating-leaved plant cluster, the floating input plant cluster, and the submerged plant cluster were harvested 6 times randomly in the range of 500 m^2^ for each sampling point with an underwater sickle with a cross-sectional area of 0.785 m^2^, and the plant was uprooted and then washed, removing residual sticks and other impurities. The fresh weight of each plant was weighed and the frequency, coverage area, and coverage were recorded (visual method). All the plants, together with the roots, were dug and washed, and the plant depth was measured [24].

## 3. Results and Discussion

### 3.1. Characteristics of Aquatic Plants in the Poyang Lake

**Figure 1:**
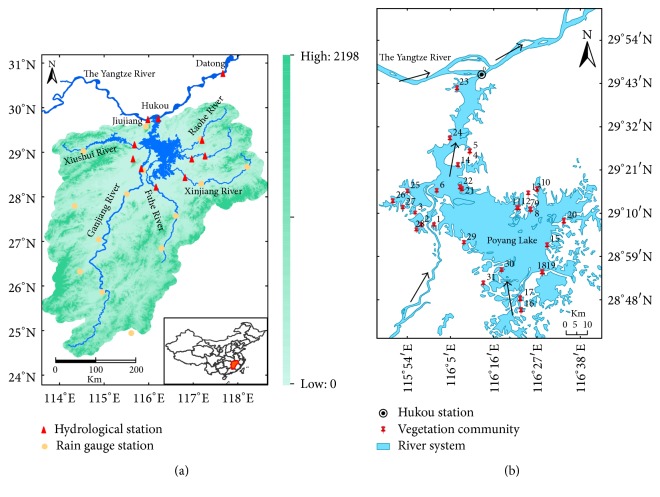
Location of the Poyang Lake river basin (a) and concerned distribution of the vegetation chart (b).

There were 43 species of aquatic plants in the lake area which belonged to 37 genera and 22 families according the survey in 2013 (see Figure 1 and Table 1). And it could be found that there were 16, 13, and 9 species, accounting for 37.21%, 30.23%, and 20.93% of the total species for the amphibians, emergent plants, and submerged plants, respectively. However, there were only 2 and 3 species for floating input plants and floating-leaved plants in the Poyang Lake, accounting for 4.65% and 6.98% of the total species.

It could be seen that the distribution of all kinds of plants was different; the most extensive degrees of various classes were* Carex *spp.,* Eleocharis tuberosa *(Roxb.) Roem. et Schult.,* Polygonum *L., and* Nymphoides peltatum *(Gmel.) O. Kuntze. Most of the species were distributed in a large area or in a continuous distribution in the wetland or near shore of the lake, which were widely distributed and had strong adaptability to adversity. Most of the plants in the whole lake were in vegetative period.

The survey recorded 22 families, 37 genera, and 43 aquatic plant species; the number of species was significantly lower than the survey of aquatic plant species number in Poyang Lake [3], which may be because the latter sampling sites in the Poyang Lake were a bit too much. The results of this study were compared with the results of the survey of 28 families, 56 genera, 95 species, and 3 varieties of aquatic vegetation in the Poyang Lake [18]. The number of species was different and the reason may be the strength of this investigation was not enough, and the accuracy of traditional field survey research method was much lower than that of TM Landsat remote sensing technology. In view of this survey was only a single survey in autumn and was limited to water habitat, lakeside vegetation was not a detailed investigation, and, therefore, the actual distribution of Poyang Lake aquatic plant species should be lower than the findings of Guan et al. [3] and Jian et al. [18].

### 3.2. Community Types and Habitats of Major Aquatic Plants in the Poyang Lake

The species of aquatic plants, the spatial structure, and ecological environment of the community were listed in Table 2 in the Poyang Lake. There were 31 main plant clusters in the Poyang Lake.

The amphibians mainly distributed in the water depth of 0.5–2.5 m meters of the beach in the flood season. The main species were amphibious plants that grew in both shallow and wetlands. There were 16 kinds of amphibians in the Poyang Lake, of which the most important and wide distribution was* Carex *spp., the least distribution was* Sambucus chinensis *Lindl.,* Isachne globosa *(Thunb.) Kuntze,* Microcarpaea minima *(Koen.) Merr., and* Oryza rufipogon *Griff.

Emergent plants were located in the 12–15 m elevation of the shoal, and the water depth was generally 0.5–3.5 m in the flood season. The main species were plants that had only the base or lower part of the plant to be submerged in water, but the upper part of the plant was quite out of water. There were 13 kinds of emergent plants in the Poyang Lake, of which the most important and wide distribution was* Eleocharis tuberosa *(Roxb.) Roem. et Schult*., *and the least distribution was* Aeschynomene indica *Linn.,* Typha angustifolia *L*., Monochoria vaginalis *(Burm.f.) Presl,* Rotala rotundifolia *(Buch.-Ham) Koehne, and* Myriophyllum tuberculatum *Roxburgh.

Floating-leaved plants were located 11–13 meters above the lake bottom, where the lake water level was generally 2.5–4.5 meters in the flood season. The main species were some plants rooted in the lake, but the leaves were floating on the surface of the water. Moreover, a large number of submerged plants could also be found in this plant belt, such as* Vallisneria natans *(Lour.) Hara and* Hydrilla verticillata *(L.f.) Royle. There were 3 kinds of floating-leaved plants in the Poyang Lake, of which the most important and wide distribution was* Nymphoides peltatum *(Gmel.) O. Kuntze, and the least distribution was* Trapella sinensis *Oliv.

Submerged plants distributed in the 9–12 m elevation of the lake, and the lake water depth was generally 3.5–6.5 meters in the flood season. The main species were some of the plants immersed in water, such as* Hydrilla verticillata *(L.f.) Royle,* Ceratophyllum demersum *L. There were 9 kinds of submerged plants in the Poyang Lake, of which the most important and wide distribution was* Ceratophyllum demersum *L. and* Vallisneria natans *(Lour.) Hara, and the least distribution was* Blyxa japonica *(Miq.) Maxim.,* Myriophyllum spicatum *L., and* Utricularia vulgaris *L.

The distribution area for floating input plant was very small, and the floating input plant was mainly located in small patches or sporadic distribution in the bay. There were 2 kinds of floating input plant in the Poyang Lake, of which the most important and wide distribution was* Eichhornia crassipes *(Mart.) Solms, and the least distribution was* Hydrocharis dubia *(Bl.) Back.

The main habitat types of aquatic macrophytes in the Poyang Lake were summarized in Table 3. Among the amphibians, the coverage of the typical* Carex *spp. cluster was generally 40–80%, and the plant height was about 25–100 cm. But in the lower edge of the distribution area, the growth of the* Carex *spp. cluster was delayed, and the plant coverage and height were relatively small. At the upper edge of the distribution area, with the increase of the elevation, the plant growth rate began to decline, and the coverage reduced accordingly. The plant height of the typical* Phragmites communis *Trin. cluster in the emerged plant was 56–250 cm, and the coverage was lower and was only 10–15%. The height of submerged plant cluster was 15–70 cm, and its coverage was large and up to 90%. The coverage of floating input plant cluster was about 50–95% and the plant height was 10–55 cm. The cluster coverage of floating-leaved plants was about 60% and plant height was 3–55 cm.

### 3.3. Quantitative Characteristics of Aquatic Plant Communities in the Poyang Lake

The number of aquatic plants in the lake area was 67, and the total of the aquatic plants' fresh weight was 1519.41 t. Among them, the* Carex *spp.,* Eleocharis tuberosa *(Roxb.) Roem. et Schult*.,* and emergent plants were 919.66 t, while the submerged plants and floating-leaved plants were up to 599.75 t. The distribution of amphibians accounted for 58.21% of the total number of samples, and there were 14.93%, 16.42%, and 10.45% of the total plants number for the emergent plants, submerged plants, and floating-leaved plants in the Poyang Lake, respectively. It could be found that the number of amphibians was the dominant plant species in the Poyang Lake, and their quantity and percentage of the total biomass were predominant, which were far more than the other three life forms (Table 4).

**Figure 2:**
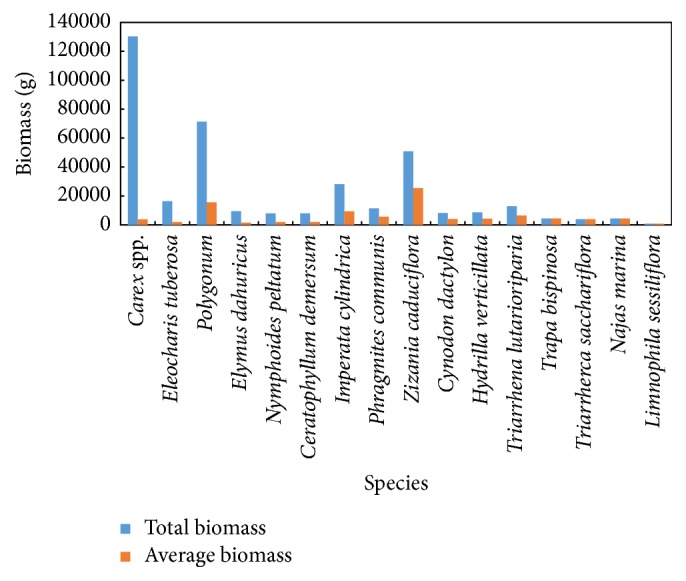
The total biomass and average biomass of the typical aquatic macrophyte's association types in the Poyang Lake.

Among the 16 typical cluster types, the total biomass was the highest in the* Carex *spp. cluster, followed by the* Polygonum *L. cluster, the* Zizania caduciflora *(Turcz. ex Trin.) H.-M. cluster, and the* Imperata cylindrica *(Linn.) Beauv cluster (Figure 2). This was in contrast to 1998 aquatic vegetation survey [25], the difference was large, and the proportion of* Vallisneria natans *(Lour.) Hara and* Hydrilla verticillata *(L.f.) Royle was the largest, while the proportion of* Carex *spp. and* Polygonum *L. was very low. In the amphibians,* Carex *spp. cluster was the dominant species, and the distribution number was 34. In the emergent plants,* Zizania caduciflora *(Turcz. ex Trin.) H.-M. cluster, and* Phragmites communis *Trin. cluster distribution were in large quantities, each for 2. In the submerged plants, the distribution of* Ceratophyllum demersum *L. cluster was the largest, with 4 species. In the floating-leaved plants, the distribution of* Nymphoides peltatum *(Gmel.) O. Kuntze cluster was the largest, with 4 species. This pattern of distribution was associated with its reproductive strategies. The vast majority of grassland vegetation in the Poyang Lake interrupted their breeding during the summer season, which was suitable for plant growth during the flood season. Therefore, the plant with vigorous regenerative ability, for example,* Carex *spp*.,* had become the most dominant species in the Poyang Lake grassland vegetation and* Carex* spp. were submerged by flood and became the carp and crucian carp spawning attachment. cluster in Poyang Lake was not only beneficial to grazing firewood and green manure, but also conducive to the reproduction of grass-laying fish sand the growth of herbivorous fish. The production of carp and crucian carp in the fishery of the Poyang Lake was about half of the total output, which was closely related to the seasonal succession of* Carex *spp. [26].

## 4. Conclusions

In this study, the distribution of aquatic vegetation and associated community diversity and biomass in the Poyang Lake were investigated with the aim of getting information for the monitoring and protection of aquatic plants in the Poyang Lake. Some interesting conclusions were obtained as follows:

1. Nine species of aquatic plants were found in the Poyang Lake and* Carex *spp. dominated absolutely with multi grads 58 in the Poyang Lake. Most of the aquatic plant species were amphibians and a small number of the species were found on the beach floating plants or submerged plants or emergent plants.
2. The vegetation of the Poyang Lake scattered in different areas which could be divided into 31 major plant communities and 5 plant zones including amphibian, emergent, floating-leaved, submerged, and floating input. The majority of aquatic vegetation distribution area in the Poyang Lake was* Carex *spp. cluster.
3. There were 67 aquatic plants in the Poyang Lake, and the total of the aquatic plants' fresh weight was 1519.41 t. Moreover, it could be found that the amphibians were the dominant plant species in the Poyang Lake.

## Figures and Tables

**Table 1:** The composition of macrophytes in the Poyang Lake (September 7–14, 2013).

Serial number	Plant community	Phenological period	Lifestyle
1	*Sagittaria pygmaea* Miq.	Flowering fruit bearing stage	Emerged plant
2	*Trapella sinensis* Oliv.	Fruit period (florescence)	Floating-leaved plant
3	*Carex rhynchophysa* C. A. Mey.	Vegetative period	Amphibian
4	*Eichhornia crassipes* (Mart.) Solms	Florescence	Floating input plant
5	*Aeschynomene indica* Linn.	Flowering fruit bearing stage	Emerged plant
6	*Sambucus chinensis* Lindl.	Vegetative period	Amphibian
7	*Isachne globosa* (Thunb.) Kuntze	Florescence	Amphibian
8	*Limnophila sessillflora* (Vahl.) Bl.	Vegetative period	Amphibian
9	*Paspalum distichum* L.	Vegetative period	Emerged plant
10	*Hydrocharis dubia *(Bl.) Back.	Fruit period	Floating input plant
11	*Ottelia alismoides* (L.) Pers.	Flowering fruit bearing stage	Submerged plant
12	*Ludwigia adscendens* (L.) Hara	Flowering fruit bearing stage	Amphibian
13	*Juncellus serotinus*	Flowering fruit bearing stage	Amphibian
14	*Blyxa japonica* (Miq.) Maxim.	—	Submerged plant
15	*Marsilea quadrifolia* L.	Vegetative period	Amphibian
16	*Nephrolepis cordifolia *(L.) Presl	Fruit period	Amphibian
17	*Carex *spp.	Florescence	Amphibian
18	*Microcarpaea minima* (Koen.) Merr.	Vegetative period	Amphibian
19	*Oryza rufipogon* Griff.	Flowering fruit bearing stage	Amphibian
20	*Myriophyllum spicatum* L.	Vegetative period	Submerged plant
21	*Typha angustifolia* L.	Fruit period	Emerged plant
22	*Monochoria vaginalis* (Burm.f.) Presl	—	Emerged plant
23	*Rotala rotundifolia *(Buch.-Ham) Koehne	—	Emerged plant
24	*Imperata cylindrica* (Linn.) Beauv	Vegetative period	Amphibian
25	*Eleocharis tuberosa* (Roxb.) Roem. et Schult.	Vegetative period	Emerged plant
26	*Najas marina*	Fruit period	Submerged plant
27	*Triarrherca sacchariflora*	Vegetative period	Emerged plant
28	*Cynodon dactylon *(Linn.) Pers.	Vegetative period	Amphibian
29	*Zizania caduciflora *(Turcz. ex Trin.) H.-M.	Vegetative period	Emerged plant
30	*Hydrilla verticillata* (L.f.) Royle	Vegetative period	Submerged plant
31	*Utricularia aurea* Lour.	Florescence	Submerged plant
32	*Ceratophyllum demersum* L.	Vegetative period	Submerged plant
33	*Vallisneria natans* (Lour.) Hara	Flowering fruit bearing stage	Submerged plant
34	*Utricularia vulgaris *L.	Flowering fruit bearing stage	Submerged plant
35	*Polygonum* L.	Vegetative period	Amphibian
36	*Trapa bispinosa* Roxb.	Flowering fruit bearing stage	Floating-leaved plant
37	*Phragmites communis* Trin.	Vegetative period	Emerged plant
38	*Triarrhena lutarioriparia*	Vegetative period	Amphibian
39	*Alternanthera Philoxeroides* (Mart.) Griseb.	Florescence	Emerged plant
40	*Nymphoides peltatum* (Gmel.) O. Kuntze	Florescence	Floating-leaved plant
41	*Limnophila heterophylla* (Roxb.) Benth.	Flowering fruit bearing stage	Emerged plant
42	*Myriophyllum tuberculatum *Roxburgh	Fruit period	Emerged plant
43	*Elymus dahuricus* Turcz.	Vegetative period	Amphibian

**Table 2:** The aquatic plant's association types in the Poyang Lake.

Serial number	Plant community
1	*Vallisneria natans* (Lour.) Hara + *Carex *spp*. + Limnophila sessiliflora* (Vahl.) Bl. + *Ceratophyllum demersum* L.
2	*Carex *spp*. + Elymus dahuricus* Turcz. +*Vallisneria natans* (Lour.) Hara + *Myriophyllum tuberculatum *Roxburgh + *Nymphoides peltatum* (Gmel.) O. Kuntze + *Ceratophyllum demersum* L. + *Limnophila heterophylla* (Roxb.) Benth. + *Eleocharis tuberosa* (Roxb.) Roem. et Schult.
3	*Carex *spp.
4	*Alternanthera philoxeroides *(Mart.) Griseb. + *Paspalum distichum* L. + *Paspalum distichum* L.-*Alternanthera philoxeroides* (Mart.) Griseb.+*Typha angustifolia* L.-*Alternanthera philoxeroides* (Mart.) Griseb.
5	*Aeschynomene indica* Linn. + *Juncellus serotinus + Oryza rufipogon* Griff.
6	*Carex *spp*. + Polygonum* L.
7	*Marsilea quadrifolia* L.
8	*Paspalum distichum* L.
9	*Eichhornia crassipes* (Mart.) Solms + *Nymphoides peltatum* (Gmel.) O. Kuntze
10	*Trapa bispinosa* Roxb. + *Microcarpaea minima* (Koen.) Merr. + *Isachne globosa* (Thunb.) Kuntze + *Carex *spp.
11	*Eleocharis tuberosa* (Roxb.) Roem. et Schult. + *Ludwigia adscendens* (L.) Hara + *Elymus dahuricus* Turcz.-*Carex *spp. + *Myriophyllum spicatum* L.-*Hydrilla verticillata* (L.f.) Royle + *Hydrilla verticillata* (L.f.) Royle-*Vallisneria natans* (Lour.) Hara.
12	*Carex rhynchophysa* C. A. Mey.
13	*Blyxa japonica* (Miq.) Maxim. + *Juncellus serotinus + Sagittaria pygmaea* Miq.-*Hydrilla verticillata* (L.f.) Royle + *Monochoria vaginalis *(Burm.f.) Presl*-Rotala rotundifolia* (Buch.-Ham) Koehne.
14	*Eichhornia crassipes* (Mart.) Solms + *Nymphoides peltatum* (Gmel.) O. Kuntze + *Marsilea quadrifolia* L. + *Alternanthera philoxeroides* (Mart.) Griseb.-*Eichhornia crassipes* (Mart.) Solms.
15	*Hydrocharis dubia *(Bl.) Back.+ *Triarrherca sacchariflora + Carex *spp*. + Hydrilla verticillata* (L.f.) Royle + *Vallisneria natans* (Lour.) Hara + *Ceratophyllum demersum* L. + *Najas marina-Eleocharis tuberosa *(Roxb.) Roem. et Schult. + *Carex *spp*.-Triarrherca sacchariflora.*
16	*Vallisneria natans* (Lour.) Hara + *Nymphoides peltatum* (Gmel.) O. Kuntze + *Carex* spp. *+ Limnophila heterophylla* (Roxb.) Benth. + *Ottelia alismoides* (L.) Pers. + *Trapa bispinosa* Roxb.-*Ottelia alismoides* (L.) Pers. + *Utricularia aurea* Lour. + *Hydrocharis dubia* (Bl.) Back.-*Trapa bispinosa* Roxb. + *Carex rhynchophysa* C. A. Mey. + *Trapella sinensis* Oliv. + *Sagittaria pygmaea* Miq. + *Eleocharis tuberosa* (Roxb.) Roem. et Schult.
17	*Carex *spp*. + Nymphoides peltatum* (Gmel.) O. Kuntze + *Eleocharis tuberosa* (Roxb.) Roem. et Schult. + *Zizania caduciflora *(Turcz. ex Trin.) H.-M. + *Elymus dahuricus* Turcz. + *Phragmites communis* Trin. + *Ceratophyllum demersum* L.
18	*Carex *spp.
19	*Elymus dahuricus* Turcz.
20	*Carex *spp*. + Eleocharis tuberosa* (Roxb.) Roem. et Schult. *+ Nymphoides peltatum* (Gmel.) O. Kuntze + *Eleocharis tuberosa* (Roxb.) Roem. et Schult.-*Polygonum* L.
21	*Carex *spp*. + Polygonum* L. + *Eleocharis tuberosa* (Roxb.) Roem. et Schult.-*Polygonum* L. + *Carex *spp.-*Eleocharis tuberosa* (Roxb.) Roem. et Schult.
22	*Carex *spp*. + Polygonum* L.
23	*Carex *spp*. + Polygonum* L. + *Cynodon dactylon *(Linn.) Pers.
24	*Carex *spp*. + Elymus dahuricus* Turcz. + *Phragmites communis* Trin.-*Cynodon dactylon* (Linn.) Pers.
25	*Ceratophyllum demersum* L. + *Nymphoides peltatum* (Gmel.) O. Kuntze + *Imperata cylindrica* (Linn.) Beauv + *Carex *spp*. + Hydrilla verticillata* (L.f.) Royle + *Vallisneria natans* (Lour.) Hara + *Eleocharis tuberosa* (Roxb.) Roem. et Schult.
26	*Carex *spp.
27	*Carex *spp*. + Eleocharis tuberosa* (Roxb.) Roem. et Schult. + *Imperata cylindrica* (Linn.) Beauv.
28	*Carex *spp*. + Trapa bispinosa* Roxb. + *Ceratophyllum demersum* L. + *Utricularia aurea* Lour. + *Triarrhena lutarioriparia + Triarrherca sacchariflora.*
29	*Hydrilla verticillata* (L.f.) Royle + *Najas marina + Eleocharis tuberosa* (Roxb.) Roem. et Schult. + *Ceratophyllum demersum* L. + *Carex *spp*. + Nymphoides peltatum* (Gmel.) O. Kuntze + *Vallisneria natans* (Lour.) Hara.
30	*Najas marina + Trapa bispinosa* Roxb. + *Carex *spp*. + Zizania caduciflora *(Turcz. ex Trin.) H.-M. + *Triarrhena lutarioriparia. *
31	*Polygonum* L. + *Limnophila sessiliflora* (Vahl.) Bl. + *Utricularia vulgaris *L*. + Carex *spp*. + Eleocharis tuberosa* (Roxb.) Roem. et Schult. + *Elymus dahuricus* Turcz. + *Polygonum* L.

**Table 3:** The habitat profiles of main association types in macrophyte communities in the Poyang Lake.

Serial number	Cluster type	Sampling date	Multiplicity-cluster degree	Height (cm)	Number of plants	Coverage (%)
1	*Limnophila sessiliflora* (Vahl.) Bl.	2013/9/7	4, 3	15–25	82	60
		2013/9/14	3, 3	3-3	78	60
2	*Limnophila heterophylla* (Roxb.) Benth.	2013/9/7	5, 4	37–45	144	90
		2013/9/13	4, 4	3–10	135	50
3	*Carex *spp.	2013/9/7	4, 5	45–70	294	80
		2013/9/7	3, 3	50–80	170	40
		2013/9/8	5, 5	50–100	3600	100
		2013/9/13	4, 5	25–65	300	70
4	*Vallisneria natans* (Lour.) Hara	2013/9/7	5, 3	45–70	26	90
		2013/9/12	5, 3	15–30	20	90
5	*Eleocharis tuberosa* (Roxb.) Roem. et Schult.	2013/9/7	3, 3	20–50	850	30
		2013/9/11	5, 5	30–85	1000	90
		2013/9/14	1, 4	18–35	350	10
6	*Phragmites communis* Trin.	2013/9/7	2, 2	60–250	80	15
		2013/9/14	1, 1	56–70	36	10
7	*Zizania caduciflora *(Turcz. ex Trin.) H.-M.	2013/9/7	4, 5	60–120	260	60
		2013/9/13	5, 4	70–168	144	85
8	*Eleocharis tuberosa* (Roxb.) Roem.et Schult.-Polygonum L.	2013/9/8	3, 3	3–60	240	40
		2013/9/9	5, 5	25–49	1000	95
9	*Alternanthera philoxeroides* (Mart.) Griseb.	2013/9/9	5, 4	10–50	900	100
		2013/9/9	5, 5	42–70	672	95
10	*Paspalum distichum* L.-*Alternanthera philoxeroides* (Mart.) Griseb.	2013/9/9	4, 3	10–25	1600	70
11	*Typha angustifolia* L.-*Alternanthera philoxeroides *(Mart.) Griseb.	2013/9/9	4, 3	80–200	25	60
12	*Paspalum distichum* L.	2013/9/9	4, 5	32–55	600	80
		2013/9/10	4, 5	20–35	1000	100
13	*Aeschynomene indica* Linn.	2013/9/9	5, 3	180–200	23	85
14	*Oryza rufipogon* Griff.	2013/9/9	5, 5	70–120	160	100
15	*Juncellus serotinus*	2013/9/9	2, 1	90–120	23	30
		2013/9/10	5, 3	20–60	850	90
16	*Carex *spp.-*Eleocharis tuberosa* (Roxb.) Roem. et Schult.	2013/9/9	4, 3	43–79	640	60
17	*Polygonum* L.	2013/9/9	3, 4	4–10	2000	40
		2013/9/10	5, 5	20–45	1000	90
		2013/9/14	3, 3	20–72	57	60
18	*Cynodon dactylon *(Linn.) Pers.	2013/9/10	5, 5	6–10	420	90
19	*Marsilea quadrifolia* L.	2013/9/10	3, 2	15–25	368	40
20	*Eichhornia crassipes* (Mart.) Solms	2013/9/10	3, 2	45–55	21	80
		2013/9/11	5, 4	12–25	170	95
21	*Isachne globosa* (Thunb.) Kuntze	2013/9/10	5, 4	20–35	700	80
22	*Elymus dahuricus* Turcz.-*Carex *spp.	2013/9/10	2, 5	70–120	150	30
23	*Ludwigia adscendens* (L.) Hara	2013/9/10	5, 4	20–40	108	95
24	*Carex rhynchophysa* C. A. Mey.	2013/9/10	3, 3	100–150	750	50
25	*Sagittaria pygmaea* Miq.-*Hydrilla verticillata* (L.f.) Royle	2013/9/10	3, 3	10–17	23	40
26	*Phragmites communis* Trin.-*Cynodon dactylon *(Linn.) Pers.	2013/9/11	3, 3	30–90	95	40
27	*Nymphoides peltatum* (Gmel.) O. Kuntze	2013/9/11	4, 3	3–8	560	60
		2013/9/13	4, 3	35–55	30	60
28	*Imperata cylindrica* (Linn.) Beauv	2013/9/11	5, 5	35–65	2560	90
		2013/9/12	5, 4	60–110	960	80
		2013/9/12	5, 4	60–110	960	80
29	*Triarrhena lutarioriparia*	2013/9/12	5, 4	70–165	650	85
		2013/9/13	5, 4	70–98	570	80
30	*Triarrherca sacchariflora*	2013/9/12	5, 5	80–103	200	95
31	*Hydrocharis dubia *(Bl.) Back.	2013/9/12	3, 3	10–15	30	50
32	*Ottelia alismoides* (L.) Pers.	2013/9/13	3, 4	15	20	80
		2013/9/13	4, 4	15–30	20	80
33	*Hydrocharis dubia* (Bl.) Back.-*Trapa bispinosa* Roxb.	2013/9/13	4, 4	10–15	96	70
34	*Carex rhynchophysa* C. A. Mey.	2013/9/13	4, 4	60–100	350	60
35	*Sagittaria pygmaea* Miq.	2013/9/13	5, 3	10–20	52	75
36	*Nephrolepis cordifolia *(L.) Presl	2013/9/14	5, 5	13–25	500	95
		2013/9/14	5, 5	13–20	400	90
37	*Sambucus chinensis* Lindl.	2013/9/14	2, 2	10–27	100	40
38	*Elymus dahuricus* Turcz.	2013/9/7	1, 2	10–30	340	10
		2013/9/11	3, 3	20–70	40	50
		2013/9/14	5, 4	62–131	280	80

**Table 4:** Number of the association area and standing crop (fresh weight) in the total vegetation area and standing crop in the Poyang Lake.

Serial number	Plant community	Quadrat number	Existing quantity (t)			
			Fresh weight	Fresh weight range	Dry weight	Dry weight range
1	*Imperata cylindrica* (Linn.) Beauv	3	38.33	35–45	10.26	9.18–10.8
2	*Eleocharis tuberosa* (Roxb.) Roem. et Schult.	6	94.08	11.5–380	6.285	4.62–11.34
3	*Eleocharis tuberosa* (Roxb.) Roem. et Schult.-*Polygonum* L.	2	137.5	75–200	21.345	20.65–22.04
4	*Najas marina*	1	35	—	25.53	—
5	*Triarrherca sacchariflora*	1	40	—	9.32	—
6	*Cynodon dactylon* (Linn.) Pers.	1	40	—	5.8	—
7	*Zizania caduciflora *(Turcz. ex Trin.) H.-M.	2	99.76	9.52–190	104.08	6.46–201.7
8	*Hydrilla verticillata* (L.f.) Royle	2	42.5	35–50	6.195	3.61–8.78
9	*Utricularia aurea* Lour.	1	50	—	15.28	—
10	*Elymus dahuricus* Turcz.	3	148.05	14.15–340	23.86333	5.99–55
11	*Ceratophyllum demersum* L.	4	80	35–150	6.7775	3.07–10.08
12	*Vallisneria natans* (Lour.) Hara	2	29.75	4.5–55	3.86	3.24–4.48
13	*Utricularia vulgaris *L.	1	25	—	3.6	—
14	*Polygonum* L.	6	34.11333	10.34–50	6.218333	4.6–9.71
15	*Trapa bispinosa* Roxb.	2	57.5	55–60	6.35	2.93–9.77
16	*Phragmites communis* Trin.	1	120	—	15.43	—
17	*Phragmites communis* Trin.- *Cynodon dactylon* (Linn.) Pers.	1	20	—	9.4	—
18	*Triarrhena lutarioriparia*	1	27.03	—	11.73	—
19	*Limnophila sessiliflora* (Vahl) Blume	1	12.8	—	4.29	—
20	*Carex *spp.	20	72.992	24.84–360	8.7425	4.22–16.96
21	*Carex *spp.-*Eleocharis tuberosa* (Roxb.) Roem. et Schult.	1	35	—	8.53	—
22	*Nymphoides peltatum* (Gmel.) O. Kuntze	5	280	20–280	43.4	3.3–43.4

## References

[1] Jun H., Xiao-hong G., Guo-feng L. (2008). Aquatic macrophytes in East Lake Taihu and its interaction with water environment. *Journal of Lake Sciences*.

[2] Luo J., Li X., Ma R. (2016). Applying remote sensing techniques to monitoring seasonal and interannual changes of aquatic vegetation in Taihu Lake, China. *Ecological Indicators*.

[3] Guan S., Lang Q., Zhang B. (1987). Aquatic vegetation of Poyang Lake. *Acta Hydrobiologica Sinica*.

[4] Lan C., Shen Y., Wang B. (2010). Investigation of aquatic plants and benthic macroinvertebrates of lakes in Inner Mongolia-Xinjiang Plateau. *Journal of Lake Sciences*.

[5] Mânzatu C., Nagy B., Ceccarini A., Iannelli R., Giannarelli S., Majdik C. (2015). Laboratory tests for the phytoextraction of heavy metals from polluted harbor sediments using aquatic plants. *Marine Pollution Bulletin*.

[6] Rezania S., Taib S. M., Md Din M. F., Dahalan F. A., Kamyab H. (2016). Comprehensive review on phytotechnology: heavy metals removal by diverse aquatic plants species from wastewater. *Journal of Hazardous Materials*.

[7] Massicotte P., Bertolo A., Brodeur P., Hudon C., Mingelbier M., Magnan P. (2015). Influence of the aquatic vegetation landscape on larval fish abundance. *Journal of Great Lakes Research*.

[8] Churchill R. T. J., Schummer M. L., Petrie S. A., Henry H. A. L. (2016). Long-term changes in distribution and abundance of submerged aquatic vegetation and dreissenid mussels in Long Point Bay, Lake Erie. *Journal of Great Lakes Research*.

[9] Shen G., Liao J., Guo H., Liu J. (2015). Poyang Lake wetland vegetation biomass inversion using polarimetric RADARSAT-2 synthetic aperture radar data. *Journal of Applied Remote Sensing*.

[10] Liao J., Shen G., Dong L. (2013). Biomass estimation of wetland vegetation in Poyang Lake area using ENVISAT advanced synthetic aperture radar data. *Journal of Applied Remote Sensing*.

[11] Han X., Chen X., Feng L. (2014). Four decades of winter wetland changes in Poyang Lake based on Landsat observations between 1973 and 2013. *Remote Sensing of Environment*.

[12] Daoust R. J., Childers D. L. (1998). Quantifying aboveground biomass and estimating net aboveground primary production for wetland macrophytes using a non-destructive phenometric technique. *Aquatic Botany*.

[13] Silva T. S. F., Costa M. P. F., Melack J. M. (2010). Assessment of two biomass estimation methods for aquatic vegetation growing on the Amazon Floodplain. *Aquatic Botany*.

[14] Zhao D., Jiang H., Yang T., Cai Y., Xu D., An S. (2012). Remote sensing of aquatic vegetation distribution in Taihu Lake using an improved classification tree with modified thresholds. *Journal of Environmental Management*.

[15] Zhang L., Yin J., Jiang Y., Wang H. (2012). Relationship between the hydrological conditions and the distribution of vegetation communities within the Poyang Lake National Nature Reserve, China. *Ecological Informatics*.

[16] Wang X., Xu L., Wan R., Chen Y. (2016). Seasonal variations of soil microbial biomass within two typical wetland areas along the vegetation gradient of Poyang Lake, China. *Catena*.

[17] Guan S., Lang Q., Zhang B. (1987). Biomass of macrophytes of the Poyang Lake with suggestions of its rational exploitation. *Acta Hydrobiologica Sinica*.

[18] Jian Y., Li R., Wang J., Chen J. (2001). Aquatic plant diversity and remote sensing of the beach vegetation in lake Poyang. *Acta Phytoecologica Sinica*.

[19] Peng Y., Jian Y., Li R. (2003). Community diversity of aquatic plants in the lakes of Poyang Plain District of China. *Journal of Central South Forestry University*.

[20] Zhang Z., Chen X., Xu C.-Y., Hong Y., Hardy J., Sun Z. (2015). Examining the influence of river-lake interaction on the drought and water resources in the Poyang Lake basin. *Journal of Hydrology*.

[21] Vereecken H., Baetens J., Viaene P., Mostaert F., Meire P. (2006). Ecological management of aquatic plants: effects in lowland streams. *Hydrobiologia*.

[22] Cook C. D. K. (1974). *Water Plants of the World*.

[23] Chen H. (1980). The structure and dynamicsofaquatic vascular plant communities in DongLake, Wuhan. *Oceanologia et Limnologia Sinica*.

[24] Hua W., Shuiping C., Peihong C. (2011). Investigation of aquatic macrophytes in lakes of Beihu Watershed of East Lake area in Wuhan, in the autumn of 2009. *Journal of Lake Sciences*.

[25] Li W., Liu G., Xiong B. (2004). The restoration of aquatic vegetation in lakes of Poyang Lake nature reserve after catastrophic flooding in 1998. *Journal of Wuhan Botanical Research*.

[26] Zhang B., Wang J. (1982). Preliminary opinions on the protection and utilization of natural resources in Poyang Lake. *Freshwater Fisheries*.

